# Why do we still give prophylactic topical antibiotics after intravitreal injections?

**DOI:** 10.1038/s41433-026-04812-7

**Published:** 2026-08-18

**Authors:** Hashem Abu Serhan

**Affiliations:** Department of Ophthalmology, Hamad Medical Corporation, Doha, Qatar

**Keywords:** Retinal diseases, Education, Health care economics

Intravitreal anti-vascular endothelial growth factor (anti-VEGF) injections are among the most frequently performed procedures in modern medicine, with millions administered annually worldwide. Although post-injection infectious endophthalmitis is a devastating complication, its incidence remains exceptionally low (approximately 0.02–0.05%). Nevertheless, topical antibiotics continue to be prescribed routinely by many clinicians despite an overwhelming body of evidence demonstrating no protective benefit. This disconnect raises an important question: why do we continue prescribing a treatment that appears ineffective, costly, and potentially harmful?

The evidence has been remarkably consistent over the past decade. Storey et al. evaluated 117,171 intravitreal injections and found that post-injection topical antibiotics did not reduce the incidence of either suspected or culture-positive endophthalmitis. In fact, antibiotic use was associated with a trend toward a higher risk of infection [1].

Similarly, Morioka et al. analysed 147,440 anti-VEGF injections from multiple Japanese centres and reported endophthalmitis rates of 0.005% in patients who received no antibiotics compared with 0.009–0.012% in those receiving antibiotic prophylaxis, with no statistically significant differences between groups. Notably, nine of the ten patients who developed endophthalmitis had received topical antibiotics, and several cultured organisms were resistant to the administered agents [2].

Comparable findings have been reported in Europe. Torres-Costa et al. compared more than 33,000 injections before and after discontinuation of routine topical antibiotics and found no difference in endophthalmitis incidence (0.0337% vs. 0.0428%; *p* = 0.675), concluding that eliminating antibiotic prophylaxis had no adverse effect on patient safety [3].

Prospective evidence reaches the same conclusion. Tanaka et al. prospectively evaluated over 18,500 consecutive intravitreal injections performed using meticulous 0.25% povidone-iodine irrigation but without topical antibiotics. Remarkably, no cases of infectious endophthalmitis occurred, demonstrating that careful antisepsis alone is sufficient for maintaining extremely low infection rates [4].

Perhaps the strongest evidence comes from systematic reviews. Reibaldi et al. pooled data from 60 studies encompassing 639,391 injections and found that eyes receiving topical antibiotics had an estimated endophthalmitis incidence of 9 per 10,000 injections, compared with 3 per 10,000 injections in eyes receiving no antibiotics [5]. Likewise, Bande et al. analysed 276,774 injections and demonstrated a 70% higher risk of endophthalmitis in patients receiving topical antibiotics (RR 1.70; 95% CI 1.08–2.66) [6]. Although these findings likely reflect confounding rather than a true causal relationship, they clearly demonstrate that antibiotic prophylaxis offers no measurable clinical benefit.

The biological explanation is straightforward. Endophthalmitis develops when microorganisms are introduced into the eye during needle penetration. Antibiotic drops administered after the procedure cannot eliminate organisms that have already entered the vitreous cavity. In contrast, povidone-iodine rapidly reduces conjunctival bacterial load within seconds, has broad-spectrum antimicrobial activity, and has never been associated with clinically significant bacterial resistance, making it the single most effective prophylactic intervention.

Beyond the absence of efficacy, repeated antibiotic exposure contributes to antimicrobial resistance. Moss et al. demonstrated high rates of fluoroquinolone resistance among conjunctival flora from patients undergoing repeated intravitreal injections, with resistance observed in 42% of isolates to ciprofloxacin, 39% to levofloxacin, and 22% to gatifloxacin [7]. Similarly, investigators at the Bascom Palmer Eye Institute documented a decline in susceptibility of coagulase-negative staphylococci to newer fluoroquinolones from 96.6 to 65.4% over approximately one decade [8]. Furthermore, Kim and Toma showed that repeated exposure to topical antibiotics during serial injections selected for multidrug-resistant conjunctival flora, with 81.8% of isolates resistant to at least three antibiotics and 67.5% resistant to five or more antibiotics after one year [9]. These findings are particularly concerning given that many patients require lifelong anti-VEGF therapy.

Cost is another compelling reason to abandon this practice. Anti-VEGF therapy already imposes substantial financial and logistical burdens on patients and caregivers because of frequent clinic visits. Tabano et al. recently demonstrated considerable six-month treatment-related costs across Europe and North America, with economic burden increasing as injection frequency increased [10]. Prescribing an ineffective medication after every injection only amplifies this burden.

Economic analyses also fail to support antibiotic prophylaxis. Sharifi et al. showed that povidone-iodine is highly cost-effective for preventing endophthalmitis, whereas topical antibiotics are not. Their model demonstrated that commonly prescribed ophthalmic antibiotics would need to become several-fold less expensive simply to achieve cost-equivalence with povidone-iodine, even under favourable assumptions regarding efficacy [11].

Given such overwhelming evidence, why do many clinicians continue prescribing topical antibiotics? The answer likely lies in historical practice patterns, medicolegal concerns, and therapeutic inertia rather than science. Earlier surgical traditions favoured postoperative antibiotics, and this approach was extrapolated to intravitreal injections before high-quality evidence became available. However, current evidence consistently demonstrates that meticulous povidone-iodine antisepsis—not postoperative antibiotics—is the cornerstone of endophthalmitis prevention.

As the number of intravitreal injections continues to grow worldwide, ophthalmology should continue embracing evidence-based practice. Routine prophylactic topical antibiotics do not reduce endophthalmitis, increase antimicrobial resistance, add unnecessary costs and treatment burden, and should no longer have a role in standard intravitreal injection protocols.
